# Psychiatric comorbidity in epilepsy and difficulties in treatment: A case report

**DOI:** 10.1192/j.eurpsy.2022.1173

**Published:** 2022-09-01

**Authors:** M. Fernández Fariña, F. Mayor Sanabria, C. Regueiro Martín-Albo, M.E. Expósito Durán, A. Francos Ajona

**Affiliations:** Hospital Clínico San Carlos, Instituto De Psiquiatría Y Salud Mental, Madrid, Spain

**Keywords:** epilepsy, comorbidity, Depression, Impulsivity

## Abstract

**Introduction:**

We present the case of a 36-year-old male with depressive states, impulsive traits and fits of anger (including episodes of self and heteroagressiveness) since early childhood, in the context of traumatic family history and the beginning of an epileptic disease. These symptoms have been maintained over the years, in addition to other variable and recurrent symptoms, such as severe anxiety, somatizations or serious depressive symptoms.

**Objectives:**

To highlight the possible influence of epilepsy in the course of mental illnesses, especially depression, as well as the increased difficulty in management.

**Methods:**

We collected the complete medical history of a patient with an important history of mental health in addition to epileptic disease since childhood and we carried out a review of the comorbidity between these diseases and their treatment.

**Results:**

The epileptic disease of our patient may have influenced the behavioural alterations and the depressive symptoms since childhood, as well as the personality traits with aggressiveness and impulsiveness. There is an added difficulty in treating this case given the possible interactions between antiepileptic and antidepressant medications.

**Conclusions:**

This case highlights the importance of taking into account the influence of this comorbidity on the prognosis of patients. Knowing the interactions and side effects of drugs is essential for good clinical management.

**Disclosure:**

No significant relationships.

